# How Does AI Trust Foster Innovative Performance Under Paternalistic Leadership? The Roles of AI Crafting and Leader’s AI Opportunity Perception

**DOI:** 10.3390/bs15081064

**Published:** 2025-08-05

**Authors:** Qichao Zhang, Feiwen Wang, Ganli Liao, Miaomiao Li

**Affiliations:** Business School, Beijing Information Science and Technology University, Beijing 102206, China; zhangqichao@bistu.edu.cn (Q.Z.); 2023020786@bistu.edu.cn (F.W.); 20192277@bistu.edu.cn (M.L.)

**Keywords:** AI trust, paternalistic leadership, AI crafting, innovative performance, leader’s AI opportunity perception, trait activation theory

## Abstract

As artificial intelligence (AI) becomes increasingly embedded in organizational development, understanding how leadership shapes employee responses to AI is critical for fostering workplace innovation. Drawing on trait activation theory, this study develops a theoretical model in which employee AI trust enhances innovative performance through AI crafting. Paternalistic leadership serves as a situational moderator, while the leader’s AI opportunity perception functions as a higher-order moderator. A three-wave survey was conducted with 523 employees from 14 AI-intensive manufacturing firms in China. Results show that the interaction between AI trust and paternalistic leadership positively predicts both AI crafting and innovative performance. In addition, AI crafting mediates the effect of the interaction term on innovative performance. Furthermore, the leader’s AI opportunity perception moderates this interactive effect: when this perception is high, the positive impact of AI trust and paternalistic leadership on AI crafting is significantly stronger; when it is low, the effect weakens. These findings contribute to the literature by clarifying the situational and cognitive conditions under which AI trust promotes innovation, thereby extending trait activation theory to AI-enabled workplaces and offering actionable insights for leadership development in the intelligent era.

## 1. Introduction

With the increasing integration of AI into daily lives, trust has become a crucial component in in interactions between humans and AI (Glikson & Woolley, 2020). AI trust is a fundamental prerequisite for individuals to accept and collaborate with AI (Chowdhury et al., 2022; Sanders et al., 2019). However, the establishment of trust between humans and AI remains a significant challenge (Afroogh et al., 2024). According to the 2025 Global Artificial Intelligence Survey, 54% of global respondents express caution toward AI, with only 46% indicating a willingness to trust the technology. In emerging economies such as China, the level of trust in AI is notably lower, standing at just 57% (Gillespie et al., 2025). The underlying reasons are manifold, including the complexity of AI algorithms and the requirement for employees to share personal data, which raises concerns about privacy and security (Siau & Wang, 2020; Stephanidis et al., 2019). Under these circumstances, AI technologies may fall short of fostering employees’ innovative performance if AI trust is lacking (Kong et al., 2024). Considering these far-reaching implications, it is critical to investigate the situational factors that can effectively activate employees’ AI trust, ultimately fostering innovation.

Emerging studies highlight the critical role of leadership styles in overcoming employees’ trust barriers toward AI and fostering high-quality human–AI collaboration (Odugbesan et al., 2023; Yin et al., 2024). However, leadership theories commonly used in prior research may lack full cultural adaptability in Eastern enterprises (X. P. Chen et al., 2014; Liu et al., 2022). In this context, paternalistic leadership, which comprises authority, benevolence, and morality, has received increasing scholarly attention (X. P. Chen et al., 2014; B. S. Cheng et al., 2004). Trait activation theory (TAT) offers a framework for understanding the impact of paternalistic leadership on employee innovation performance. According to TAT, psychological traits are activated in appropriate situations, prompting behaviors that influence individual performance (Tett & Burnett, 2003). First, benevolent leadership fosters an open environment by encouraging trial and error, thereby providing psychological safety for employees to engage in AI-driven innovation (Li & Bitterly, 2024; W. Lin et al., 2018). Second, moral leadership enhances perceptions of organizational fairness and the ethical application of AI, promoting responsible and normative innovation practices (Lythreatis et al., 2024). Third, authoritarian leadership increases employees’ willingness to comply with AI-related decisions and strengthens consistency and execution in innovation activities (Ning et al., 2012; Zhang et al., 2021).

Furthermore, grounded in the “Trait × situation → behavior → performance” framework, we conceptualize AI crafting as a mediating mechanism (Tett et al., 2021; Judge & Zapata, 2015). Given that AI crafting entails inherent risks and uncertainties, employees are unlikely to initiate such efforts unless they both trust AI and perceive the organizational atmosphere as supportive and psychologically safe (Barclay et al., 2022; Dierdorff & Jensen, 2018). Paternalistic leadership is recognized for forming such an environment, thereby facilitating the effective translation of AI trust into sustained crafting efforts (X. P. Chen et al., 2014; Erben & Güneşer, 2008). In addition, TAT emphasizes that only salient and behaviorally relevant situational cues can effectively elicit trait-driven responses (Carter et al., 2024; Yan et al., 2025). Although paternalistic leadership conveys socio-emotional signals rooted in cultural norms, it may not always provide sufficiently clear cues about how employees should engage with AI. Only when leaders recognize the value of AI are they more inclined to create conditions that encourage constructive employee transformation (Chatterjee et al., 2022; W. Li et al., 2024). Thus, the leader’s AI opportunity perception (LAIOP) may be a key boundary condition enabling paternalistic leadership to activate employees’ AI trust and promote AI crafting and innovation.

Our research offers several contributions to the literature on paternalistic leadership, AI trust, AI crafting, AI opportunity perception, and employee innovation. First, drawing on TAT, we demonstrate that paternalistic leadership is a potent situational cue that activates employees’ AI trust, thereby facilitating their innovative performance. Second, we identify AI crafting as a core behavioral mechanism, linking AI trust to innovation. This enriches job crafting theory with a context-sensitive lens on employee adaptation to AI. Third, we uncover LAIOP as a critical boundary condition: only when LAIOP is high can paternalistic leadership and AI trust effectively trigger AI crafting. It deepens our understanding of how LAIOP can amplify the effect of paternalistic leadership, revealing how such leadership can be adapted to unlock employee innovation in the era of AI.

## 2. Theoretical Background and Research Hypothesis

### 2.1. Trait Activation Theory

TAT provides a theoretical framework for understanding how personality traits are expressed and translated into behavior in the workplace (Tett & Guterman, 2000). As the theoretical scope of TAT continues to broaden, researchers have increasingly applied it to stable psychological constructs beyond traditional personality traits, such as psychological capital and prosocial value motives, demonstrating its versatility and explanatory power in accounting for emerging constructs in organizational behavior (Cai et al., 2019; Kim et al., 2013). Additionally, trait-relevant cues vary by their situational source and are typically classified into task-level, social-level, and organizational-level subsystems (Tett & Burnett, 2003). Among these, leadership is considered a prototypical organizational-level cue (Luria et al., 2019). Due to its critical role in eliciting trait-relevant behaviors, TAT has been increasingly applied to leadership research (Phaneuf et al., 2016). In our study, paternalistic leadership and LAIOP constitute the organizational situational cues that activate employees’ AI trust as the focal trait. Specifically paternalistic leadership supplies the socio-emotional signals that can activate employees’ AI trust, and LAIOP intensifies the clarity of leadership signals, reinforcing employees’ belief in engaging with AI. Exploring how these situational cues activate employees’ AI trust offers valuable insights into enhancing their innovation performance in the era of AI transformation.

### 2.2. AI Trust, Paternalistic Leadership, and Innovative Performance

AI trust is defined as a psychological tendency in which individuals hold a positive attitude and confidence during their interaction with AI, based on the belief that AI has the ability to help them achieve specific goals (Glikson & Woolley, 2020). Drawing on TAT, we proposed that paternalistic leadership serves as a salient situational cue that activates employees’ AI trust, thereby enhancing innovative performance (Tett & Burnett, 2003). Paternalistic leadership embodies benevolence, morality, and authority, which is typically enacted in organizational contexts characterized by hierarchical order and personalized power structures (B. S. Cheng et al., 2004). Firstly, benevolent leadership plays an inspirational role by fostering a psychologically safe environment. It allows employees to feel more secure when engaging in tasks involving AI (Afroogh et al., 2024; Chan, 2017). As a result, employees are more likely to view the organization as supportive of technological experimentation, which boosts their willingness to engage in AI-assisted innovation efforts (Li & Bitterly, 2024). Secondly, integrity, fairness, and responsibility of moral leaders help to build a foundation of trust for the ethical use of AI (Engelbrecht et al., 2017). This normative signal reduces employees’ subjective concerns regarding the ethical and legal risks associated with technological applications, thereby increasing their willingness to utilize AI (Lowman, 2025). On this basis, employees’ AI trust reinforces their positive evaluations of its technical capabilities, ultimately improving the quality and acceptability of innovation outcomes. Thirdly, authoritarian leadership provides clear goals and rigid standards, offering directional support for employees to translate their trust in AI into productive behaviors (L. Chen et al., 2024; Zhang et al., 2021). When employees possess high levels of AI trust, the clarity and structure provided by authoritarian leaders can further encourage the use of AI to enhance task efficiency and goal attainment. Even under tightly regulated conditions, employees may still generate meaningful innovative outcomes. Therefore, we propose the following:

**H1.**  *The interaction between AI trust and paternalistic leadership positively influences innovative performance. The higher the level of paternalistic leadership, the stronger the positive effect of AI trust on innovative performance.*

### 2.3. The Mediating Role of AI Crafting

AI crafting refers to the proactive efforts employees make to adjust the role of AI in their work processes (W. Li et al., 2024). This includes optimizing the division of tasks between humans and AI, reshaping modes of collaboration, and reconstructing the cognitive meaning of AI to fully leverage its empowering potential. Contingent on TAT, individuals exhibit trait-consistent behaviors when situations activate those traits (Tett & Guterman, 2000). Firstly, benevolent leadership provides employees with emotional support, resources, and growth opportunities, which boosts their adaptability and initiative during technological change (W. Lin et al., 2018). Employees are more likely to view AI as a partner and readily accept and cooperate with AI (Choung et al., 2023; Shao et al., 2024). The stability provided by benevolent leadership reduces employees’ uncertainty about AI. This reinforces the belief that AI is conducive to both them and organization (Siau & Wang, 2020). Secondly, moral leadership sets clear ethical guidelines for AI use by demonstrating principled behavior and establishing clear standards (Brown & Treviño, 2006; Fehr et al., 2015). This ethical approach builds employees’ AI trust, making them more willing to use AI responsibly in their tasks (Avey et al., 2012). Thirdly, for authoritarian leadership, its clear directives and structured environment reduce uncertainty and anxiety about AI, enabling employees to form stable routines (Pellegrini & Scandura, 2008; Zheng et al., 2020). For those who already trust AI, this clarity further reinforces their willingness to cooperate with the system (Zhang et al., 2021). Additionally, focusing on collective goals and shared responsibility helps employees cognitive reframing and promoting proactive behavior related to AI for organizational benefit. Therefore, we propose the following:

**H2.**  *The interaction between AI trust and paternalistic leadership positively influences AI crafting. The higher the level of paternalistic leadership, the stronger the positive effect of AI trust on AI crafting.*

Furthermore, existing research has demonstrated that job crafting significantly enhances employee innovation and mediates the relationship between trust and innovative outcomes (Bindl et al., 2019; Khan et al., 2021; Tho, 2022). Extending this logic, our study argues that under the joint influence of AI trust and paternalistic leadership, AI crafting is the primary pathway driving innovation performance. Firstly, through task crafting, employees can delegate repetitive tasks to AI, freeing up cognitive and time resources to focus on complex problem-solving and experimentation. This shift encourages employees to explore new approaches and generate innovative ideas, ultimately enhancing individual innovation (Bindl et al., 2019; B. Lin et al., 2017). Secondly, integrating AI into collaborative processes enables employees who trust AI to refine their interaction patterns with both AI and colleagues, such as through data sharing and joint decision-making. This reduces information silos and promotes knowledge complementarity, facilitating the integration of diverse perspectives to improve innovation efficiency (Kong et al., 2023; Shao et al., 2024). Thirdly, frequent engagement with AI helps employees redefine their work goals and values, viewing AI as a tool to enhance their own capabilities. This cognitive reframing broadens their role boundaries and stimulates more innovative thinking (B. Lin et al., 2017). Therefore, we propose the following:

**H3.**  *AI crafting mediates the effect of the interaction between AI trust and paternalistic leadership on employees’ innovation performance.*

### 2.4. The Moderating Role of Leader’s AI Opportunity Perception

LAIOP captures the extent to which leaders recognize and comprehend the potential opportunities that AI technologies present (G. Xu et al., 2023). This construct reflects how leaders perceive AI’s potential benefits for career advancement, skill development, and workplace improvements (Schwesig et al., 2023). Current research on AI opportunity perception has primarily concentrated on the employee perspective (G. Xu et al., 2023; Schwesig et al., 2023). However, leaders play a more pivotal role in shaping the organizational climate for technological support, guiding subordinates’ adaptive behaviors, and fostering employee innovation (Bunjak et al., 2022). Importantly, LAIOP alone is unlikely to directly drive employees’ proactive AI crafting. As W. Li et al. (2024) demonstrated, only leader’s explicit AI-related behaviors create a trickle-down effect on subordinates’ corresponding actions. This influence depends on being combined with specific leadership behaviors and employees’ AI trust (Epitropaki et al., 2017; Tett & Burnett, 2003).

According to TAT, multiple consistent cues can more effectively trigger the influence of individual traits on behavior (Luria et al., 2019; Tett et al., 2021). High LAIOP enables paternalistic leadership to leverage its management potential more effectively, enhancing employees’ cognition and behavioral tendencies toward AI. Specifically, benevolent leadership fosters a supportive environment for AI adaptation, encouraging trial and error while offering psychological backing. This allows employees to confidently experiment with AI-driven job crafting (Li & Bitterly, 2024). Moral leadership alleviates employees’ concerns regarding the ethical risks of AI technology. This, in turn, encourages their active participation in human–machine collaboration and innovation initiatives (Varma et al., 2023). And authoritarian leadership, by emphasizing AI’s strategic importance and allocating resources, clarifies technological goals and expectations, thus increasing employees’ trust-based adoption of AI (Zeng, 2020). In contrast, when LAIOP is low, the effectiveness of paternalistic leadership in facilitating employees’ AI-related behaviors is greatly diminished. Benevolent leaders may lack the technical direction to support AI collaboration; moral leaders may not provide the necessary ethical guidance; and authoritarian leaders may not promote or support AI implementation. As a result, employees receive insufficient support and significance for AI, even if they themselves are willing to trust and adopt the technology. Therefore, we propose the following:

**H4.**  *There is a three-way interaction effect between LAIOP, paternalistic leadership, and AI trust on AI crafting. Specifically, LAIOP positively moderates the moderating effect of paternalistic leadership on the relationship between AI trust and AI crafting.*

Additionally, high LAIOP means viewing AI as a critical driver of organizational development, which makes their paternalistic behaviors more technology-oriented and strategic. Such leaders are better at recognizing the value of AI trust in influencing employee behavior. They turn employees’ trust in technology into motivation through strategic support, cultural influence, and guidance (Baron, 2006). As a result, employees are more likely to proactively engage in AI crafting, thus aligning their actions with organizational innovation goals and enhancing individual innovation performance. In this context, paternalistic leadership not only activates employees’ AI trust but also strengthens the entire “trust-behavior-performance” pathway by facilitating intentional and systematic AI crafting. Conversely, in a low LAIOP context, even paternalistic leaders may send negative or ambiguous signals about AI, undermining employees’ motivation to act on their trust in the technology. The absence of encouragement and role modeling reduces psychological safety, and traditional management approaches prevail over AI-driven support and direction. Thus, employees become less enthusiastic about experimenting with tasks, collaborating, or reshaping their work perceptions, resulting in lower levels of AI crafting and diminished innovation performance. Therefore, we propose the following:

**H5.**  *LAIOP moderates the mediating effect of paternalistic leadership on the relationship between AI trust, AI crafting, and innovative performance. Specifically, the higher the LAIOP, the stronger the activating effect of paternalistic leadership on the “AI trust (+) → AI crafting (+) → innovative performance” pathway.*

**Figure 1:**
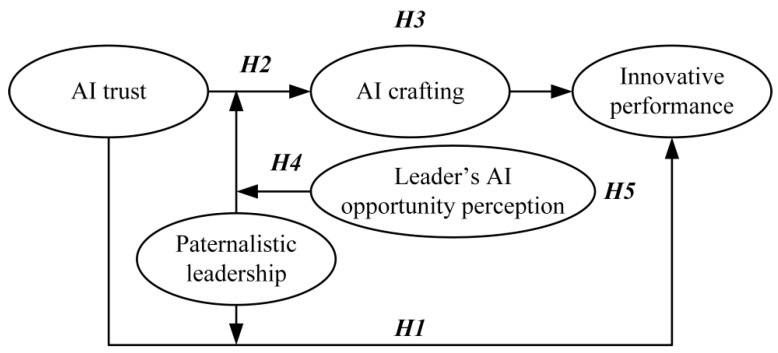
Theoretical model.

As shown in Figure 1, we draws on TAT and previous research to propose a higher-order moderation model (Arain et al., 2020; Liao et al., 2024). This modeling approach reflects an emerging perspective in leadership and technology research that highlight the importance of complex situational factors in shaping innovative outcomes.

## 3. Methods

### 3.1. Sample and Procedures

This study draws on data from 14 manufacturing firms in Beijing, Shanghai, Tianjin, and Guangdong, covering sectors such as automotive, machinery, electronics, communication equipment, and chemicals. These firms, which have made notable progress in AI-driven intelligent transformation, are considered representative of broader industrial trends. They utilize advanced AI applications in key areas, including product development, production processes, and supply chain management. For example, automotive companies use AI-powered computer vision systems to detect defects in vehicle parts and components with high accuracy, and AI is also employed to predict potential equipment failures in factories. Machinery firms apply generative AI to optimize the design of machine components, improving their weight, strength, and aerodynamics, and AI is used to enhance material supply chains by forecasting demand and managing inventory. The survey focused on employees working in research and development as well as intelligent manufacturing departments. Data collection proceeded in three stages. First, the research team worked with HR managers at each firm to define the respondent pool, ensuring sample relevance and methodological rigor. Second, to enhance response quality, participants received online briefings explaining the study’s objective, emphasizing voluntary participation, and assuring anonymity and academic use of all data. Third, questionnaires were distributed via internal email systems or We Chat workgroups, with modest monetary incentives offered at each wave (Time 1: CNY 5; Time 2: CNY 10; Time 3: CNY 15) to improve response rates. To minimize common method bias, this study adopted a three-wave data collection design. Respondents created a unique identification code based on the last four digits of their national ID number to ensure accurate data matching across time points while protecting personal privacy. At Time 1, employees self-reported their levels of AI trust, paternalistic leadership, leader’s AI opportunity perception, and demographic characteristics. A total of 658 questionnaires were distributed, and 624 valid responses were collected. At Time 2, participants assessed their AI crafting behaviors, yielding 567 valid responses. At Time 3, employees evaluated their own innovative performance, resulting in 523 valid responses. The overall response rate across the three waves was 79.48%. Among the final sample, 47.4% were male and 52.6% female; 32.3% were under 28 years old; 47.4% were aged 28–33; 16.4% were aged 33–38; and 3.8% were over 38. In terms of education, 2.9% held a junior college degree or below; 29.3% had a bachelor’s degree; 62.3% had a master’s degree; and 5.5% held a doctorate. Regarding tenure, 14.7% had less than one year of work experience; 49.3% had 1–5 years; 23.9% had 5–10 years; and 12.0% had more than 10 years.

### 3.2. Measures

This study strictly adhered to standard translation procedures by employing a translation and back-translation process of well-established scales. To ensure linguistic and conceptual equivalence, three professors in business administration and two bilingual translators reviewed the translated items against the original versions. After appropriate revisions and refinements, the Chinese version demonstrated satisfactory reliability and validity. All variables were measured using a five-point Likert scale (1 = strongly disagree, 5 = strongly agree). The measurement items are presented in Appendix A.

#### 3.2.1. AI Trust

Using the items from Chowdhury et al. (2022), the AI trust scale consists of 11 items. One sample item is as follows: “I have confidence in the use of AI technology”. The Cronbach’s α was 0.938.

#### 3.2.2. AI Crafting

Using the scale from W. Li et al. (2024)’s AI crafting scale, which consists of 6 items. A sample item is as follows: “When working with AI robots, I introduce new approaches on my own to improve my work”. The Cronbach’s α was 0.907.

#### 3.2.3. Paternalistic Leadership

Parental leadership was measured using the scale developed by B. S. Cheng et al. (2004), which includes three dimensions: authoritative leadership, benevolent leadership, and virtuous leadership. The scale contains 26 items. A sample item is as follows: “My supervisor is like a family member when he/she gets along with us”. The Cronbach’s α was 0.910.

#### 3.2.4. Leader’s AI Opportunity Perception

LAIOP was measured using a five-item scale adapted from Highhouse and Yüce (1996). A sample item is as follows: “My supervisor believes that the influence of enterprises applying AI can be controlled”. The Cronbach’s α was 0.870.

#### 3.2.5. Innovative Performance

This study employed the innovation performance scale developed by Janssen and Van Yperen (2004), which consists of three dimensions: idea generation, idea promotion, and idea realization, with a total of 9 items. A sample item is as follows: “I often introduce innovative ideas into the work environment”. The Cronbach’s α was 0.887.

#### 3.2.6. Controlled Variables

Based on previous research on employees’ innovative performance (Gu et al., 2015), this study includes gender, age, education, and tenure as control variables.

## 4. Results

### 4.1. Common Method Biases

Data for this study were collected at three different time points, with measures taken to ensure sample confidentiality and minimize common method bias by controlling response duration. To further examine common method bias, Harman’s single-factor test was conducted through factor analysis. The results showed that the first factor explained 17.20% of the total variance, which is below the 40% threshold commonly suggested in previous studies. Additionally, a six-factor model was tested by adding an unmeasured latent factor to the five-factor model. The analysis revealed no significant improvement in model fit (∆CFI = 0.009, ∆TLI = 0.009, ∆RMSEA = 0.013), indicating that common method bias is not a significant issue in this study.

### 4.2. Descriptive Analysis

This study conducted descriptive statistics and correlation analysis using SPSS 27.0. The means, standard deviations, and correlation coefficients of all variables are shown in Table 1. The results show that AI trust is positively correlated with AI crafting (r = 0.15, *p* < 0.01) and innovative performance (r = 0.17, *p* < 0.01). AI crafting is positively correlated with innovative performance (r = 0.16, *p* < 0.01). This provides preliminary evidence for the hypotheses of this study.

### 4.3. Confirmatory Factor Analysis

This study employed Mplus 8.3 to conduct confirmatory factor analysis. The results, as shown in Table 2, indicate that the five-factor model (χ2/df = 1.967, CFI = 0.912, TLI = 0.902, RMSEA = 0.043, SRMR = 0.056) has the most fit indices compared to other alternative models (e.g., one factor: χ2/df = 5.853, CFI = 0.556, TLI = 0.506, RMSEA = 0.096, SRMR = 0.147), Thus, the results demonstrate good discriminant validity between all the variables.

### 4.4. Empirical Test

The hierarchical regression model was constructed by SPSS 27.0. The results are shown in Table 3. For H1, Model 3 shows that the interaction between AI trust and paternalistic leadership is significant for innovative performance (β = 0.147, *p* < 0.01). For H2, Model 6 shows that the interaction between AI trust and paternalistic leadership is significant for AI crafting (β = 0.331, *p* < 0.01). Thus, H1, H2, and H3 were supported.

**Figure 2:**
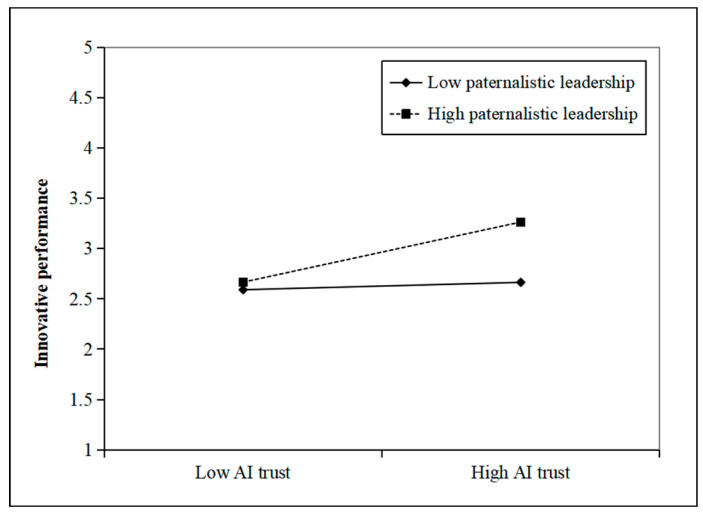
Interaction effect of AI trust and paternalistic leadership on innovative performance.

**Figure 3:**
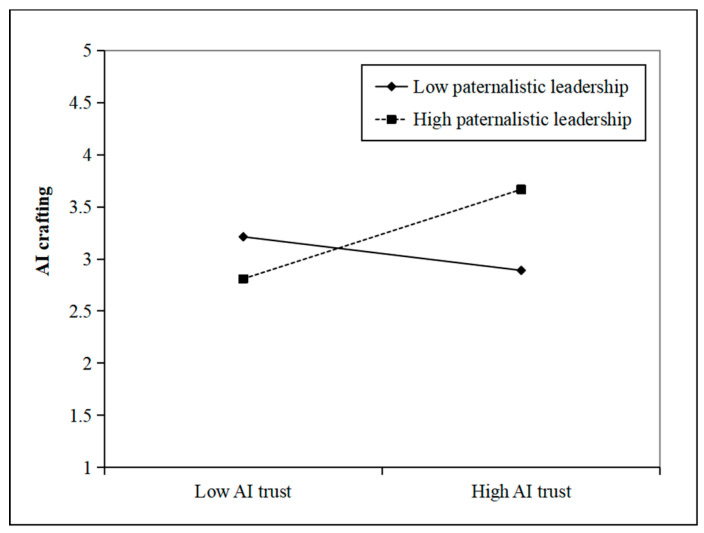
Interaction effect of AI trust and paternalistic leadership on AI crafting.

Furthermore, we used a simple slope analysis to examine the moderating effect of paternalistic leadership at different levels (Mean ± 1SD) (Aiken et al., 1991). As shown in Figure 2, compared to low paternalistic leadership (Mean − 1SD), high paternalistic leadership (Mean + 1SD) more strongly increased the positive effect of AI trust on innovative performance. In addition, as shown in Figure 3, compared to low paternalistic leadership (Mean − 1SD), high paternalistic leadership (Mean + 1SD) more strongly increased the positive effect of AI trust on AI crafting. Therefore, H1 and H2 were further verified.

In addition, bootstrap analysis (=5000 times) was conducted in the Process plug-in to further test the moderated mediating effect. The results are presented in Table 4. It shows that if the paternalistic leadership is low, then the effect is −0.020 (CI = [−0.045, −0.001]), which does not include 0, and if paternalistic leadership is high, then the effect is 0.052 (95% CI = [0.018, 0.092]), which does not include 0. However, the effect of differences between the high and low levels is 0.072 (95% CI = [0.025, 0.128]), which does not include 0. The moderated mediating effect of paternalistic leadership is significant. Therefore, H3 was verified.

Additionally, this study examines H4, which proposes the direct influence of the three-way interaction among AI trust, paternalistic leadership, and LAIOP on AI crafting. As shown in Model 4 in Table 5, the interaction has a significant positive correlation with AI crafting (β = 0.096, *p* < 0.05). This indicates that the higher the LAIOP, the more it can aggravate the activating effect of paternalistic leadership on AI crafting in employees with AI trust. To further test H4, following the method of Dawson and Richter (2006), we obtained four conditions: (1) high paternalistic leadership (Mean + 1SD) and high LAIOP (Mean + 1SD); (2) high paternalistic leadership (Mean + 1SD) and low LAIOP (Mean + 1SD); (3) low paternalistic leadership (Mean + 1SD) and high LAIOP (Mean + 1SD); (4) low paternalistic leadership (Mean + 1SD) and low LAIOP (Mean + 1SD).

**Figure 4:**
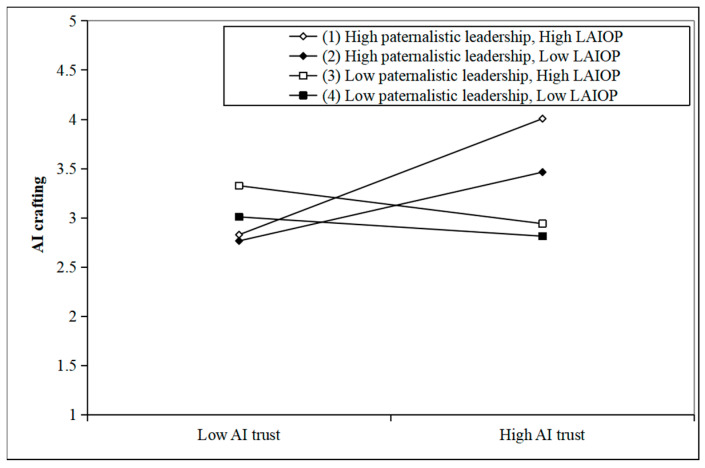
Three interaction effects of AI trust, paternalistic leadership, and LAIOP on AI crafting.

Based on these combinations, we plotted the three-way interaction effect, as shown in Figure 4. It can be seen that in the scenario of high paternalistic leadership and high LAIOP, the positive slope of the fitted curve for the influence of AI trust on AI crafting is the steepest, indicating that in this scenario, the positive influence of AI trust on AI crafting is the most significant. Thus, H4 was verified.

Continuing to use the Bootstrap method to test the dual-moderated mediation effect, the results are shown in Table 6. The results indicate that under conditions of low paternalistic leadership and low LAIOP, as well as low paternalistic leadership and high LAIOP, the 95% confidence intervals are [−0.045, 0.015] and [−0.062, 0.002], respectively. Both intervals include 0, indicating that when paternalistic leadership is low, the influence of AI trust on innovative performance through AI crafting is insignificant. Conversely, under conditions of high paternalistic leadership and low LAIOP, and high paternalistic leadership and high LAIOP, the 95% confidence intervals were [0.013, 0.083] and [0.026, 0.130], respectively. Both intervals exclude 0, and the mediation effect value for high paternalistic leadership and low LAIOP is 0.042, which is lower than the mediation effect value of 0.071 for high paternalistic leadership and high LAIOP. Therefore, it can be concluded that the influence of AI trust on innovative performance through AI crafting is most significant among employees with high paternalistic leadership and high LAIOP. Thus, H5 was further verified.

## 5. Discussion

In this paper, we attempted to investigate how paternalistic leadership interacts with AI trust to shape employee innovative performance. Building upon the chain of “Trait × situation → behavior → performance” as theorized by TAT, we proposed a theoretical framework that explicitly considered the mediating and moderating role of AI crafting and LAIOP, respectively.

Firstly, the interaction between AI trust and paternalistic leadership positively influences innovative performance. Prior research has confirmed that AI trust positively influences employee outcomes, such as AI acceptance, job performance, and productivity (Chowdhury et al., 2022; Kong et al., 2023; C. S. Lin et al., 2025). These findings provide solid support for our research, highlighting the positive impact of AI trust. In addition, the three dimensions of paternalistic leadership have been proven to have a positive effect as a situation factor (Karakitapoğlu-Aygün et al., 2020; Zhang et al., 2021). Specifically, benevolent leadership provides psychological safety for innovation, moral leadership promotes fairness and ethical application of AI, and authoritarian leadership enhances compliance and execution in innovative activities (Chan, 2017; Gu et al., 2015; Zhang et al., 2022).

Secondly, AI crafting mediates the effect of the interaction between AI trust and paternalistic leadership on innovative performance. Various AI-related factors, such as the event strength of AI introduction, AI awareness, AI dependence, and organizational AI adoption, have been shown to influence job crafting (B. Cheng et al., 2023; H. Lin et al., 2024; Khairy et al., 2025; Zhao et al., 2025). Evidence suggests that leaders’ AI crafting facilitates employees’ own AI crafting, underscoring the importance of leadership behavioral support for AI initiatives (W. Li et al., 2024). Moreover, AI crafting has demonstrated positive relationships with human–AI collaboration, AI helping, and AI engagement (Laukkarinen, 2025; W. Li et al., 2024). All of these findings provide valuable grounding for our proposition that the interaction between AI trust and paternalistic leadership may positively influence AI crafting, thereby enhancing innovation performance.

Third, LAIOP moderates the effect of the interaction between AI trust and paternalistic leadership on AI crafting. Current research predominantly examines AI opportunity perception from the perspective of ordinary employees, focusing on outcomes such as workplace wellbeing and willingness to use AI (Schwesig et al., 2023; Y. Xu et al., 2024), as well as its impact on AI adoption at the organizational level (Z. Li et al., 2025). In fact, LAIOP is also critical in shaping how employees engage with AI-related tasks and behaviors. For example, Sriharan et al. (2024) emphasize that leaders should assess and identify AI opportunities aligned with organizational priorities to ensure the success of AI transformation. And recent research on leader’s AI symbolization also emphasizes the critical role of leader’s AI opportunity perception (He et al., 2023; Hu et al., 2025). Collectively, these findings provide strong support for our conclusion regarding the pivotal role of LAIOP in facilitating effective AI-driven change.

### 5.1. Theoretical Implications

This study provides several meaningful theoretical implications. Firstly, drawing on TAT, this study illustrates how paternalistic leadership amplifies the positive impact of AI trust on innovation performance. Previous research has predominantly focused on how objective factors, such as AI system characteristics, influence AI trust (Wang & Ding, 2024; Zerilli et al., 2022). And most studies on the boundary conditions of AI trust focus on individual traits, overlooking the role of organizational-level contextual factors, and lack exploration of its mechanisms in driving innovation (Kong et al., 2024). Based on these insights, for one thing, our findings offer a new perspective on the role of paternalistic leadership in the AI era. For another, we extend the application of TAT and deepens our understanding of the organizational conditions under which AI trust functions, especially in the context of Chinese enterprises.

Secondly, this study identifies AI crafting as a central behavioral mechanism linking AI trust, paternalistic leadership, and innovative performance. Job crafting has become a hot topic in this rapidly changing era. It can be categorized into different types, including approach crafting and prevention-focused crafting (B. Cheng et al., 2023; H. Lin et al., 2024). These studies provide valuable references for our research. Based on these, we focus on AI crafting, as it uniquely addresses how employees adapt their roles to AI technologies (Laukkarinen, 2025; W. Li et al., 2024). This focus allows us to explore the distinct ways in which AI reshapes work processes, a perspective that has not been fully explored in existing job crafting research. Furthermore, our finding expands the scope of job crafting theory in AI contexts and underscores employees’ proactive adaptation under the influence of technological trust and leadership guidance.

Thirdly, this study introduces LAIOP as a crucial contextual moderator that amplifies the effect of paternalistic leadership, enabling employees to translate AI trust into behaviors that ultimately enhance innovation performance. This study deepens our understanding of how LAIOP, particularly when combined with paternalistic leadership, enhances employees’ ability to translate AI trust into actionable behaviors that promote innovation. This finding enriches our understanding of the cognitive mechanisms behind leadership and highlights how leaders’ perceptions of the technological environment significantly influence employees’ adaptive behaviors and innovation performance.

### 5.2. Practical Implications

Firstly, organizations should prioritize building employees’ AI trust as a foundation for effective AI implementation. Our findings demonstrate that employees’ AI trust significantly enhances their willingness to adapt work practices and is a key driver of innovative performance. To achieve this, organizations can provide AI workshops and training sessions that demystify AI’s capabilities and limitations, introduce low-risk pilot projects to foster hands-on experience, and create a culture emphasizing transparency, fairness, and ethical AI use (Benbya et al., 2020).

Secondly, organizations should focus on fostering paternalistic leadership to strengthen employees’ AI trust and support their adaptation to new technologies. Leaders should be trained in empathetic communication, addressing employee concerns about AI and offering emotional support. For example, leaders could create spaces for employees to openly discuss their fears about AI, building trust and encouraging AI adoption (Yue et al., 2024). In addition, leaders should receive training on communicating AI’s ethical implications, ensuring transparency and fairness. Ethical decision-making workshops can help leaders address AI-related concerns and build trust among employees (Ringelband & Warneke, 2025; Štrukelj & Dankova, 2025).

Thirdly, organizations should emphasize developing leaders’ AI opportunities perception to enable effective AI integration. Our findings show that LAIOP is better equipped to identify AI’s potential, communicate its benefits, and reduce uncertainty for paternalistic leadership. To improve this perception, organizations should provide strategy training focused on AI’s strategic advantages. For example, continuous learning through industry conferences and AI-focused courses can keep leaders informed about emerging trends (Burke, 2021). In addition, leaders should explore how AI can be leveraged across different departments, promoting trust and driving innovation (Niederman, 2021; Ren, 2022).

### 5.3. Limitations and Future Directions

This study has several limitations that set the stage for promising future studies. Firstly, from a methodological perspective, although this study employed a three-wave questionnaire design to mitigate common method bias, the data were entirely derived from employee self-reports, which may still be subject to cognitive bias, social desirability effects, and recall inaccuracies. Therefore, future research could adopt situational experiments to better examine how the activation of traits evolves under paternalistic leadership. Additionally, future studies should test the applicability of these findings in different industries and cultural contexts. This would help to better understand how paternalistic leadership and AI adoption may vary across these diverse settings.

Secondly, from a theoretical perspective, this study adopts a TAT perspective to highlight the role of the external situation in activating individual traits. Future research could shift attention to internal cognitive and motivational states. For instance, self-determination theory may help explain how paternalistic leadership satisfies employees’ psychological needs for autonomy, competence, and relatedness, thereby fostering intrinsic motivation and adaptive engagement with AI (Do et al., 2025).

Thirdly, from a model perspective, this study integrates the three dimensions of paternalistic leadership into a unified situational construct to examine its activating effect. However, prior research suggests that authoritarian leadership may evoke psychological stress and fear among employees, whereas benevolent and moral leadership may buffer these adverse effects, indicating a potentially complex interplay among the three dimensions (Yao et al., 2023). Future research could examine how reinforcing or compensatory interactions among the three dimensions influence employees’ innovation.

## Figures and Tables

**Table 1:** Descriptive analysis of variables.

Variables	M	SD	1	2	3	4	5	6	7	8
1. Gender	1.53	0.50								
2. Age	1.92	0.80	−0.04							
3. Education	2.71	0.61	−0.05	−0.14 **						
4. Tenure	2.33	0.87	−0.02	0.69 **	−0.13 **					
5. AI trust	3.06	1.12	0.01	0.07	−0.06	0.06				
6. Paternalistic leadership	3.09	0.79	−0.05 **	−0.06	0.05	−0.08	−0.25 **			
7. AI crafting	3.31	1.09	−0.15 **	0.06	0.10 *	−0.02	0.15 **	0.10 *		
8. Innovative performance	3.10	0.96	0.05	−0.06	−0.01	−0.02	0.17 **	0.15 **	0.16 **	
9. LAIOP	2.87	0.98	0.01	0.20 **	−0.18 **	0.11 *	0.01	−0.22 **	0.04	−0.15 **

Notes: * *p* < 0.05; ** *p* < 0.01.

**Table 2:** Results of confirmatory factor analysis.

Models	Factors	χ2/df	CFI	TLI	RMSEA	SRMR
Five-factor model	T, P, L, C, I	1.967	0.912	0.902	0.043	0.056
Four-factor model 1	T + P, L, C, I	3.102	0.808	0.786	0.063	0.104
Four-factor model 2	T, P + L, C, I	2.251	0.861	0.845	0.054	0.066
Three-factor model 1	T + P + L, C, I	3.654	0.758	0.730	0.071	0.110
Three-factor model 2	T, P + L, C + I	3.224	0.797	0.774	0.065	0.081
Two-factor model 1	T + P + L + C, I	4.373	0.691	0.657	0.080	0.121
Two-factor model 2	T + P + L, C + I	3.224	0.797	0.774	0.065	0.081
One-factor model	T + P + L + C + I	5.853	0.556	0.506	0.096	0.147

Notes: T represents AI trust; C represents AI crafting; I represents innovative performance; P represents paternalistic leadership; L represents leader’s AI opportunity perception.

**Table 3:** The results of hierarchical regression model.

Variables	Innovative Performance			AI Crafting		
	Model 1	Model 2	Model 3	Model 4	Model 5	Model 6
Gender	0.045	0.044	0.149	−0.145 **	−0.146 **	−0.250 **
Age	−0.093	−0.101	−0.126	0.137 *	0.130 *	0.192 *
Education	−0.011	−0.003	−0.021	0.101 *	0.109 *	0.199 **
Tenure	0.048	0.044	0.071	−0.104	−0.107	−0.121
AI trust		0.170 **	0.149 **		0.152 **	0.119 **
Paternalistic leadership			0.213 **			0.118 *
AI trust × Paternalistic leadership			0.147 **			0.331 **
R2	0.007	0.036	0.112	0.042	0.065	0.168 **
F	0.918	3.826 **	8.100 **	5.688 **	7.202 **	14.805 **

Notes: * *p* < 0.05; ** *p* < 0.01.

**Table 4:** Bootstrap method at different levels of paternalistic leadership.

Path	Mediator				Moderated Mediation	
AI trust → AI crafting → Innovative performance	**Moderator**	**Effect**	**SE**	**95% CI**	**Index**	**(CI)**
	Low PL (M − 1SD)	−0.020	0.012	[−0.045, −0.001]		
	High PL (M − 1SD)	0.052	0.019	[0.018, 0.092]	0.045	[0.016, 0.081]
	Difference group	0.072	0.027	[0.025, 0.128]		

**Table 5:** Hierarchical regression results of the moderating effect.

Variables	AI Crafting			
	Model 1	Model 2	Model 3	Model 4
Gender	−0.145 **	−0.146 **	−0.250 **	−0.253 **
Age	0.137 *	0.130 *	0.192 *	0.162 *
Education	0.101 *	0.109 *	0.199 **	0.217 **
Job tenure	−0.104	−0.107	−0.121	−0.110
AI trust		0.152 **	0.119 **	0.144 **
Paternalistic leadership			0.118 *	0.154 **
LAIOP				0.134 **
AI trust × Paternalistic leadership			0.331 **	0.345 **
AI trust × LAIOP				0.033
Paternalistic leadership × LAIOP				0.026
AI trust × Paternalistic leadership × LAIOP				0.096 *
R2	0.042	0.065	0.168	0.183
F	5.688 **	7.202 **	14.805 **	10.396 **

Notes: * *p* < 0.05; ** *p* < 0.01.

**Table 6:** Mediating effects of at different levels of moderating variables.

Paternalistic Leadership	LAIOP	The Mediating Effects of AI Crafting	SE	Low	High
0.792 (High)	0.980 (High)	0.071	0.027	0.026	0.130
0.792 (High)	−0.980 (Low)	0.042	0.018	0.013	0.083
−0.792 (Low)	0.980 (High)	−0.024	0.016	−0.062	0.002
−0.792 (Low)	−0.980 (Low)	−0.012	0.015	−0.045	0.015

## Data Availability

The data presented in this study are available upon request from the corresponding author.

## Appendix A

The following are the survey items.

AI Trust

1. I have confidence in the use of Al technology.
2. I believe Al technology can facilitate with routine and trivial tasks through automation
3. I believe my organization will be able operate Al technology reliably or consistently without failing.
4. I believe that Al technology will consistently operate providing adequate and efficient results within a broad spectrum of processes.
5. I believe Al adoption will result in creation of new jobs.
6. I have a positive attitude towards adoption of AI.
7. I believe Al technology can help in developing new skills which will benefit my career development activities.
8. I have a positive attitude towards its impact of intra-organizational business operations
9. I believe Al will positively change employee dynamics within the organization
10. Al adoption wont result in reduced focus on human skills such as creative intellect in my job.
11. I believe Al adoption will enhance the quality of my work.

AI Crafting

1. When working with AI robots, I introduce new approaches on my own to improve my work.
2. When working with AI robots, I change minor work procedures which I think are not productive on my own.
3. When working with AI robots, I change the way I do my job on my own to make it easier for myself.
4. When working with AI robots, I rearrange the tasks or procedures of the work to collaborate with AI robots more effectively on my own.
5. When working with AI robots, I learn new knowledge and skills about AI robots on my own.
6. When working with AI robots, I try to find the ways to improve the interactions with AI robots on my own.

Paternalistic Leadership

1. My supervisor is like a family member when he/she gets along with us.
2. My supervisor devotes all his/her energy to taking care of me.
3. Beyond work relations, my supervisor expresses concern about my daily life.
4. My supervisor ordinarily shows a kind concern for my comfort.
5. My supervisor will help me when I’m in an emergency.
6. My supervisor takes very thoughtful care of subordinates who have spent a long time with him/her.
7. My supervisor meets my needs according to my personal requests.
8. My supervisor encourages me when I encounter arduous problems.
9. My supervisor takes good care of my family members as well.
10. My supervisor tries to understand what the cause is when I don’t perform well.
11. My supervisor handles what is difficult to do or manage in everyday life for me.
12. My supervisor employs people according to their virtues and does not envy others’ abilities and virtues.
13. My supervisor doesn’t take the credit for my achievements and contributions for himself/herself.
14. My supervisor does not take advantage of me for personal gain.
15. My supervisor does not use guanxi (personal relationships) or back-door practices to obtain illicit personal gains.
16. My supervisor treats all employees fairly and without favoritism.
17. My supervisor leads by example in adhering to ethical standards.
18. My supervisor asks me to obey his/her instructions completely.
19. My supervisor determined all decisions in the organization whether they are important or not.
20. My supervisor always has the last say in the meeting.
21. My supervisor always behaves in a commanding fashion in front of employees.
22. I feel pressured when working with him/her.
23. My supervisor exercises strict discipline over subordinates.
24. My supervisor scolds us when we can’t accomplish our tasks.
25. My supervisor emphasizes that our group must have the best performance of all the units in the organization.
26. We have to follow his/her rules to get things done. If not, he/she punishes us severely.

Leader’s AI Opportunity Perception

1. My supervisor believes that the adoption of artificial intelligence by enterprises is beneficial to organization.
2. My supervisor believes that the influence of enterprises applying AI can be controlled.
3. My supervisor believes that the application of artificial intelligence by enterprises can increase the likelihood of his/her personal successful career development.
4. My supervisor believes that it is the opportunity for him/her that enterprises apply artificial intelligence.
5. My supervisor believes that is is possible for him/her to gain more than lose when enterprise apply artificial intelligence.

Innovative Performance

1. I often introduce innovative ideas into the work environment.
2. I often suggest new ideas to improve work performance.
3. I proactively seek new methods, techniques, or tools for work.
4. I frequently promote innovative ideas to others and seek support.
5. I always find ways to encourage organizational members to provide new ideas.
6. I often mobilize others to support innovation.
7. I frequently transform innovative ideas into practical applications at work.
8. I am capable of proposing creative solutions to problems.
9. I frequently track and evaluate the effectiveness of the application of innovative ideas.
